# 526. Implementation of Use of Monoclonal Antibody Therapy in a Large Academic Center for the Outpatient Treatment of Covid-19: Impact on 30 Day Hospitalization Rates, ED Visits and Death

**DOI:** 10.1093/ofid/ofab466.725

**Published:** 2021-12-04

**Authors:** Azra Bhimani, Vinay Srinivasan, Stacey Weinstein, Nathan Clemons, Quanna Batiste, Shangxin Yang, Omai Garner, Tara Vijayan

**Affiliations:** 1 University of California, Los Angeles, Los Angeles, CA; 2 UCLA David Geffen School of Medicine, Los Angeles, CA

## Abstract

**Background:**

Monoclonal Antibody Therapy (MAbs) has been shown to reduce rates of ED visits and hospitalizations in patients at risk for severe Covid-19 infection in clinical trials. Since November, three Mabs received emergency use authorization: Bamlanivimab (Bam), Bamlanivimab/Etesevimab (Bam/Ete) and Casirivimab/Imdevimab (Casi/imdevi). We describe here the real-world effectiveness of implementing early MAb therapy in the outpatient setting for individuals with Covid-19 at high risk of progression.

**Methods:**

We examined the records of 808 UCLA Health patients with a confirmed positive SARS-CoV2 PCR test who were either referred for outpatient Mab therapy or received Mab treatment in the emergency department (ED) between December 10, 2020, and May 3, 2021. The primary outcome of our analysis was the combined 30-day incidence of emergency department visits, hospitalizations, or death following the date of referral. SARS-CoV2 isolates of hospitalized patients who had received Mabs were sequenced to determine the presence of variants.

**Results:**

Of 808 patients, 383 were referred for treatment but did not receive treatment, 109 received Mabs in the ED and 316 patients were treated in an outpatient setting. Composite 30-day mortality, ED visits and hospital admissions were significantly reduced in the combination therapy group (Bam/Ete or Cas/Imd) compared with monotherapy (Bam alone) or no treatment groups (aHR 0.16, 95% CI .038, .67). Significant factors associated with the composite outcome included: history of lung disease (HR 4.46, 95% CI 2.89-6.90), cardiovascular disease (HR 1.87, 95% CI 1.12-3.12), kidney disease (HR 2.04, 95% CI 1.27-3.25), and immunocompromised state (HR 3.24, 95% CI 1.02-10.26) as well as high social vulnerability index (HR 1.87, 95% CI 1.13-3.10). Over one-third of hospitalized patients who had received Mabs were confirmed to have the California variant (B.1.427/29) (Figure 1).

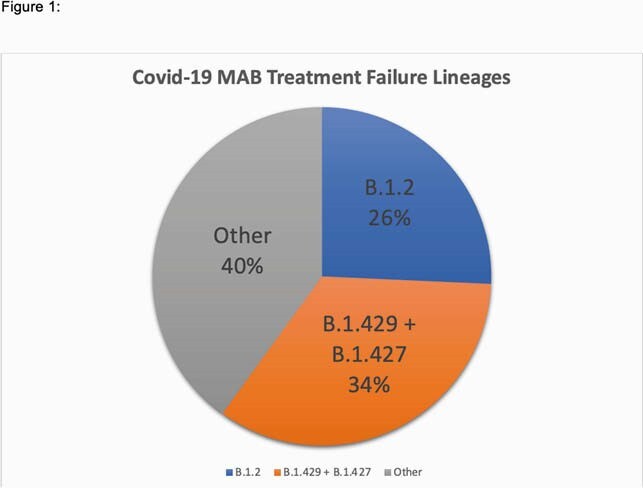

Figure 1. Covid-19 MAB Treatment Failure Lineages

**Conclusion:**

Our data show that in a real-world setting, combination monoclonal antibody therapy, not monotherapy, significantly reduced ED visits and hospital admissions, likely due to the presence of the California variants. High socioeconomic vulnerability and certain medical conditions increased risk of treatment failure.

**Disclosures:**

**Omai Garner, PhD, D(ABMM**), **Beckman Coulter** (Scientific Research Study Investigator)

